# The same modality medical image registration with large deformation and clinical application based on adaptive diffeomorphic multi-resolution demons

**DOI:** 10.1186/s12880-018-0267-3

**Published:** 2018-08-09

**Authors:** Chang Wang, Qiongqiong Ren, Xin Qin, Yi Yu

**Affiliations:** 1 School of Biomedical Engineering, Xinxiang Medical University, Xinxiang, 453003 China; 2 Xinxiang City Engineering Technology Research Center of Neurosensor and Control, Xinxiang, 453003 China

**Keywords:** Diffeomorphic, Demons, Adaptive, Large deformation, Multi-resolution

## Abstract

**Background:**

Diffeomorphic demons can not only guarantee smooth and reversible deformation, but also avoid unreasonable deformation. However, the number of iterations which has great influence on the registration result needs to be set manually.

**Methods:**

This study proposed a novel method to exploit the adaptive diffeomorphic multi-resolution demons algorithm to the non-rigid registration of the same modality medical images with large deformation. Firstly an optimized non-rigid registration framework and the diffeomorphism strategy were used, and then a similarity energy function based on the grey value was designed as registration metric, lastly termination condition was set based on the variation of this metric and iterations can be stopped adaptively. Quantitative analyses based on the registration evaluation indexes were conducted to prove the validity of this method.

**Results:**

Registration result of synthetic image and the same modality MRI and CT image was compared with those obtained by other demons algorithms. Quantitative analyses demonstrated the proposed method’s superiority. Medical image with large deformation was produced by rotational distortion and extrusion transform, and the same modality image registration with large deformation was performed successfully. Quantitative analyses showed that the registration evaluation indexes remained stable with an increase in transform strength. This method can be also applied to pulmonary medical image registration with large deformation successfully, and it showed the clinical application value. The influence of different driving forces and parameters on the registration result was analysed, and the result demonstrated that the proposed method is effective and robust.

**Conclusions:**

This method can solve the non-rigid registration problem of the same modality medical image with large deformation showing promise for diagnostic pulmonary imaging applications.

## Background

Non-rigid registration has been applied to inter-subjective registration to detect lesions and to establish a medical atlas. Comparisons of non-rigid registration algorithms have shown that those with demons based on the optical flow field theory provide superior results [1].

The demons algorithm was initially only applicable to image registration with small deformation. Therefore, many researchers have tried to improve it. In 2005, Wang and Pennec introduced the floating image gradient in the diffusion equation and proposed active demons [2, 3]. In 2006, Roglj et al. proposed symmetric demons method and demonstrated its high efficient [4]. In 2007, Vercauteren et al. applied the optimization framework of non-rigid registration to demons and proposed additive demons. In their method, non-rigid registration was equivalent to optimizing the similarity energy function, and iterations could be stopped adaptively [5]. In 2008, symmetric log-domain diffeomorphic demons algorithm was proposed, and Log Euclidean transformation was used to avoid time-consuming computations of Log spatial transformation [6]. In 2009, diffeomorphic demons was proposed, and it was shown that the final deformation is topologically invariant and that unreasonable deformations are not produced [7]. In 2010, Xu et al. introduced a regularization term and designed a new similarity energy function to ensure smooth and reversible deformations [8]. In 2012, Lei et al. proposed a new active gradient and curvature (G&C) demons algorithm using the curvature to control the deformation [9].

In recent years, some researchers have proposed non-rigid image registration methods with large deformation. In 2014, Lombaert et al. introduced the direct feature matching technique to find global correspondences between reference and moving images, and they proposed spectral log-demons method for diffeomorphic image registration with large deformation [10, 11]. In 2014, Kong et al. proposed a robust discriminative clustering method based on mutual information and used it to efficiently perform brain MRI segmentation [12]. In 2015, Zhao et al. used multilayer convolutional neural networks to determine scale and translation parameters and proposed the deep adaptive log-demons method for diffeomorphic image registration with large deformation [13]. In 2015, Yan et al. performed image registration with large deformation by combining manifold learning and diffeomorphic demons [14]. In 2016, Deng et al. proposed a data classification method based on fused fuzzy deep neural network and used it to efficiently discriminate WM, GM, and CSF [15].

Medical image registration plays an important role in diagnosing diseases and establishing a medical atlas. Large deformation may exist between reference image and moving image, and it could result in problems in non-rigid medical image registration with large deformation. This study proposes an adaptive diffeomorphic multi-resolution demons algorithm for medical image registration with large deformation. An optimized framework with non-rigid registration and the diffeomorphic deformation strategy were used, a similarity energy function based on the grey value was designed as a registration metric, and the termination condition was set based on the variation of this metric. Iterations could be stopped adaptively, and medical image registration with large deformation was performed. This method was tested using synthetic images and the same modality MRI and CT images, and the mean square error, normalized cross correlation, and structural similarity were used as evaluation indexes to verify the superiority of this method. Medical image registration with large deformation simulated by rotational distortion and extrusion was performed successfully. Quantitative analyses of the influences of different driving forces and parameters on the registration result showed that our method is effective and robust. This method can be applied to the same modality medical image registration with large deformation, making it promising for diagnostic pulmonary imaging applications.

## Methods

For the problems of the same modality medical image registration with large deformation, an adaptive diffeomorphic multi-resolution demons algorithm was proposed. An optimized framework with non-rigid registration and the diffeomorphic deformation strategy were used, a similarity energy function based on the grey value was designed, and termination conditions were set to stop iterations adaptively. We applied this method to the same modality medical image registration with large deformation and found that it shows promise for pulmonary medical image registration in clinical application.

### Additive demons

Non-rigid image registration is essentially a multi-parameter optimization problem involving mapping a moving image to a reference image. It defines an appropriate objective function as registration metric and then optimizes this metric. For reference image *F* and moving image *M*, the registration metric should be optimized to seek the best transform *t*^opt^. *t*:R^n^ *→* R^n^, *p → t*(*p*) is the mapping of pixel *p* of the moving image to pixel *t(p)* of the reference image. The definition of registration metric is the crucial step in the non-rigid registration process. The mean square deviation based on the grey value as a registration metric can be calculated using Eq. 1.

1 $$ Sim\left(F,M\circ t\right)=\frac{1}{2}{\left\Vert F-M\circ t\right\Vert}^2=\frac{1}{2\left|{\varOmega}_P\right|}\sum \limits_{P\in {\varOmega}_P}\left|F(p)-M\left(t(p)\right)\right| $$

In Eq. 1, *Ω* is the common region of *F* and *M* after registration, and ο is the transform operator. Directly minimizing the registration metric using Eq. 1 will lead to an unstable solution, and therefore, a regularization term needs to be added to restrict the geometric transform. The revised energy function *E(t)* is expressed using Eq. 2.

2 $$ E(t)=\frac{1}{\sigma_i} Sim\left(F,M\circ t\right)+\frac{1}{\sigma_t}\operatorname{Re}g(t) $$

In Eq. 2, *σ*_*i*_ is the local noise level, and *σ*_*t*_ is the regularization parameter. Cachier et al. [16] introduced the parameters c (not ruled spatial transform) and t (ruled spatial transform). Then, the new energy function is expressed using Eq. 3.

3 $$ E\left(c,t\right)={\left\Vert \frac{1}{\sigma_i}\left(F-M\circ c\right)\right\Vert}^2+\frac{1}{{\sigma_x}^2} Dist{\left(c,t\right)}^2+\frac{1}{\sigma_t}\operatorname{Re}g(t) $$

In Eq. 3, *Dist*(*c*, *t*) = ‖*c* − *t*‖,*σ*_*x*_ is the uncertainty degree between c and t. The displacement field *u* is produced using the space geometric transform, and two vectors are added directly to form a new vector. The energy function of additive demons algorithm comprised similarity measure term, deformation error term, and regularization term. The final energy function is expressed as follows:

4 $$ E(u)=\frac{1}{{\sigma_i}^2}{\left\Vert F-M\circ \left(t+u\right)\right\Vert}^2+\frac{1}{{\sigma_x}^2}{\left\Vert u\right\Vert}^2 $$

In Eq. 4, *u* is the updated displacement field, and *Dist*(*c*, *t*) = ‖*c* − *t*‖ = ‖*u*‖. By minimizing the energy function with respect to the displacement field, the final displacement field is expressed using Eq. 5.

5 $$ u(p)=-\frac{F(p)-M\circ t(p)}{{J_P}^2+\frac{{\sigma_i}^2(p)}{{\sigma_x}^2}}{J_P}^T $$

In Eq. 5, *σ*_*i*_(*p*) = |*F*(*p*) − *M* ∘ *t*(*p*)| and *J*_*P*_ =  − *∇*^*T*^*F*(*p*).

### Diffeomorphic deformation strategy

Diffeomorphic space was proposed in 2009, and it can make sure that the deformation is smooth, reversible, and topologically invariant. Diffeomorphic transform, which is based on the theory of Lie groups, is related to the exponential map of the velocity field v, that is, *ϕ* = exp(*v*), and a practical and fast approximation method with a scaling-and-squaring strategy was described as follows.

**Table:** 

Exponential *ϕ* = exp(*v*)	
Input: Velocity field v. Output: Diffeomorphic map *ϕ* = exp(*v*) Choose N such that 2^−*N*^*v* → 0 e.g., such that max ‖2^−*N*^*v*‖ ≤ 0.5 pixels Scale velocity field *ϕ* ← 2^−*N*^*v* for N times do *ϕ* ← *ϕ* ∘ *ϕ* end for	

### Algorithm implementation

The diffeomorphic deformation strategy was used for optimizing the energy function designed by additive demons. The deformation should always be topologically invariant. Rough deformation greatly influences the registration result; if it is calculated unsuitably, it is easy to fall into a local optimum. Therefore, a multi-resolution strategy was used, and rough deformation was calculated with low resolution. Then, the energy function was minimised and the best registration result was obtained, and it can avoid the local optimum.

Iterations also have great influence on the registration result. If the number of iterations is not set properly, the best deformation field cannot be obtained. The consumed time will increase with the increasing of iterations. The mean square deviation based on the grey value was designed as a registration metric, and iterations could be stopped adaptively depending on the variation of energy function. It can eliminate the influence of iterations on registration result. The convergence condition was defined by Eq. 6.

6 $$ \left\{\begin{array}{c} if\kern1.5em E(n)-E\left(n-1\right)<E\left(n-1\right)\ast stop\_ criterium\kern0.5em break\\ {} else\kern16em continue\end{array}\right. $$

When the displacement field was updated, three different demons driving forces were proposed as follows: Thirion’s primitive driving force, *J*_*P*_ =  − *∇*^*T*^*F*(*p*); Gauss–Newton-based advanced driving force, *J*_*P*_ =  − *∇*^*T*^(*M* ∘ *t*(*p*)); and symmetric driving force, *J*_*P*_ =  − (*∇*^*T*^*F* + *∇*^*T*^(*M* ∘ *t*(*p*)))/2.

The steps for each registration layer can be described as follows:

- Step 1: initialize displacement field.
- Step 2: calculate demons driving force *u(p)* and update velocity field *v.*
- Step 3: regulate deformation field using Gauss filter.
- Step 4: obtain exponential mapping of deformation field by diffeomorphic transform.
- Step 5: calculate similarity measurement function *E(t)* using Eq. 4.
- Step 6: judge convergence condition.

**Fig. 1:**
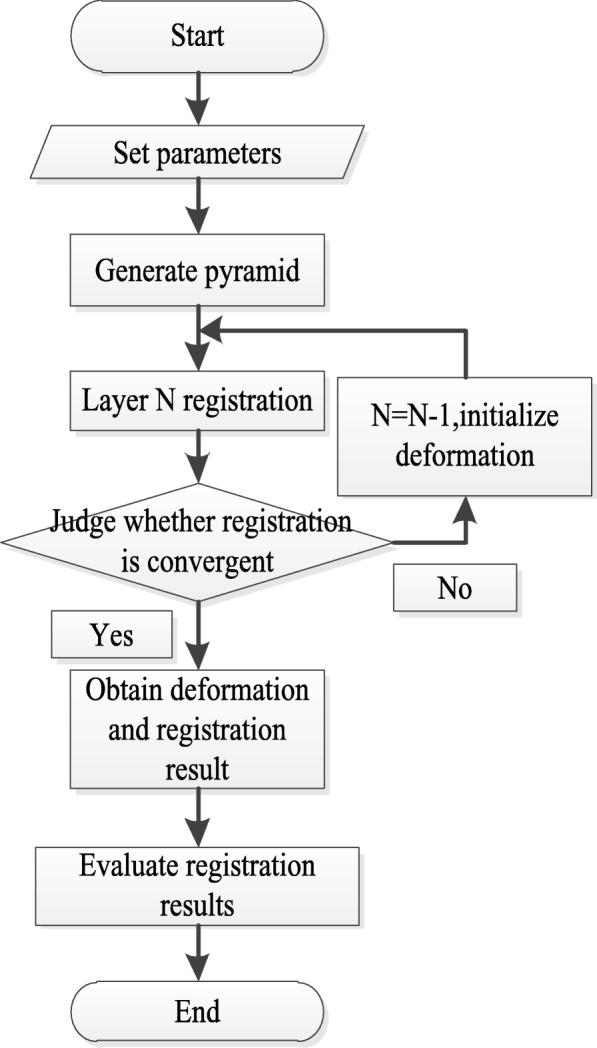
Algorithm flow

Figure 1 shows the algorithm flow of proposed method.

### Algorithm evaluation

The mean square error, normalized cross correlation, and structural similarity [17] were calculated as evaluation indexes. The mean square error is defined by Eq. 7.

7 $$ MSE=\sqrt{\frac{\sum {\left(S(x)-M(x)\right)}^2}{n}} $$

The normalized cross-correlation coefficient is defined by Eq. 8.

8 $$ CC=\frac{\sum \left(S(x)-\overline{S}\right)\left(M(x)-\overline{M}\right)}{\sqrt{\sum {\left(S(x)-\overline{S}\right)}^2}\sqrt{\sum {\left(M(x)-\overline{M}\right)}^2}} $$

Here, n is the total pixel number, S is the reference image, M is the registration result, and $\overline{S}$ and $\overline{M}$ are the average grey values of each pixel point in the reference image and registration result, respectively.

## Results

The superiority of our method was verified through comparisons with active demons, additive demons, and diffeomorphic demons. The experimental parameters were set as follows: Gaussian filter parameter *G*_*σ*_, where *σ* = 2; deformation updating step-length *σ*_*x*_ = 1.0; multi-resolution layer number *N* = 3; and convergence condition *stop_criterium* = 0.005.

### Synthetic image registration

**Fig. 2:**
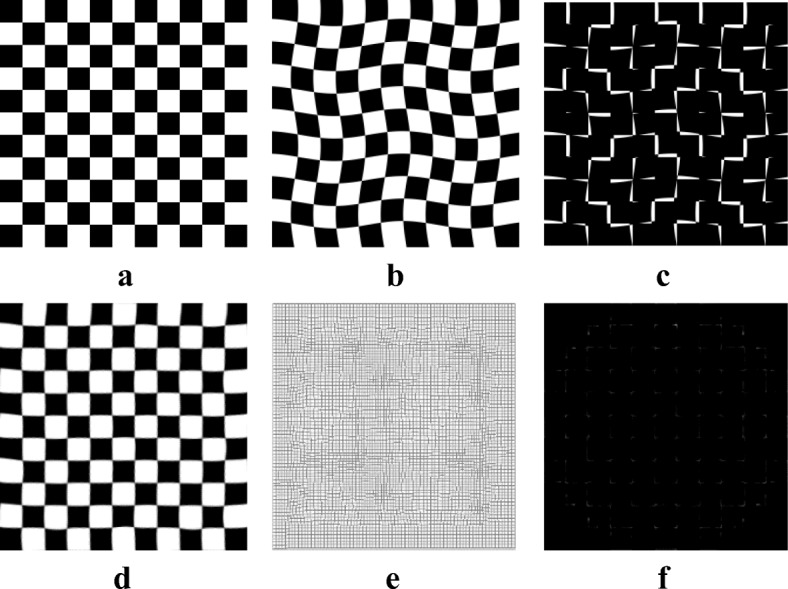
Results of synthetic checkerboard image: (**a**) reference image; (**b**) moving image; (**c**) initial difference between reference and moving image; (**d**) registration result; (**e**) final deformation; and (**f**) difference between reference and registration result

Figure 2 shows the synthetic checkerboard images that were processed to validate our method. This method can realize non-rigid image registration and estimate the deformation field effectively.

### The same modality medical image registration

**Fig. 3:**
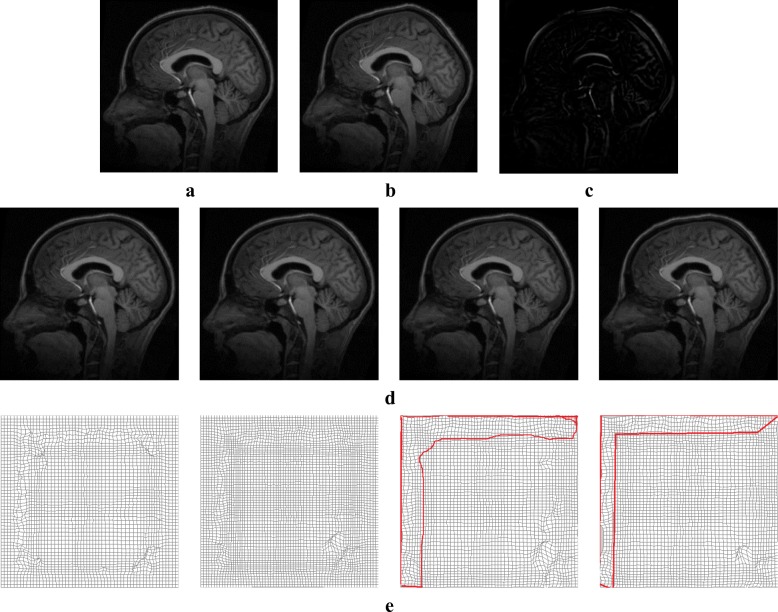
Registration result of MRI image: (**a**) reference image; (**b**) moving image; (**c**) initial difference between reference and moving images, (**d**) registration results obtained using our method, diffeomorphic demons, additive demons, and active demons, respectively, from left to right, and (**e**) final deformation field

MRI images were selected from the Simulated Brain Database (http://www.bic.mni.mcgill.ca/brainweb/) provided by the Montreal Neurology Institute of McGill University. Their size is 217 × 181 × 181, and T1-weighted MRI images were selected as reference and moving images. Figure 3(a–c) show the reference image, moving images and initial difference. Figure 3(d–e) show that our method provides the best registration result and that the deformation field is smooth and reversible. The edge of deformation field is more reasonable than that in the case of active demons and additive demons. Table 1 shows the quantitative analysis of evaluation indexes for MRI image registration. It is seen that the normalized cross-correlation coefficient and structural similarity are the highest and the mean square error is the lowest; therefore, our method is superior to active demons, additive demons, and diffeomorphic demons.

**Table 1:** Evaluation of registration result (MRI)

Experiment Method	MSE/(A.U.)	NCC/(A.U.)	Structural Similarity/(A.U.)
our method	514.7965	0.9993	0.9952
diffeomorphic demons	583.0147	0.9992	0.9937
additive demons	640.9294	0.9987	0.9906
active demons	1307.5	0.9944	0.9824

**Fig. 4:**
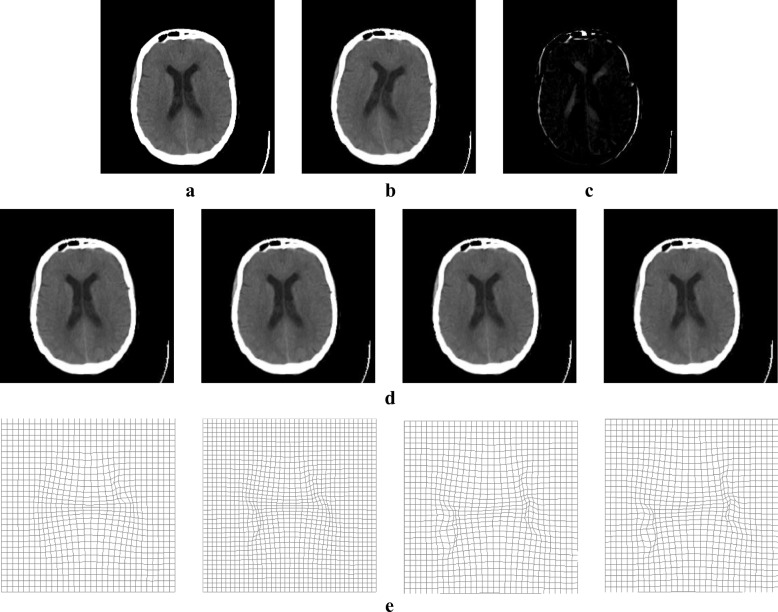
Registration result of CT image: (**a**) reference image; (**b**) moving image; (**c**) initial difference between reference and moving images; (**d**) registration results obtained using our method, diffeomorphic demons, additive demons, and active demons, respectively, from left to right; and (**e**) final deformation field

CT images were selected from the Medical Image Database (http://www.med.harvard.edu/AANLIB) provided by Harvard Medical Center, and their size is 256 × 256. Figure 4 shows the registration result of CT images, and Table 2 shows the quantitative analysis of evaluation indexes for CT image registration. Comparative analysis showed that the registration and statistical results of CT image were the same as those of the MRI image, indicating that our method can be also applicable to CT image registration.

**Table 2:** Evaluation of registration result (CT)

Experiment Method	MSE/(A.U.)	NCC/(A.U.)	Structural Similarity/(A.U.)
our method	2257.2	0.9928	0.9762
diffeomorphic demons	2578.6	0.9905	0.9619
additive demons	2377.0	0.9920	0.9635
active demons	2332.2	0.9923	0.9644

### Medical image registration with large deformation

Large deformation was produced by two types of free transforms: rotational distortion and extrusion. The deformation field was determined by the transform strength.

**Fig. 5:**
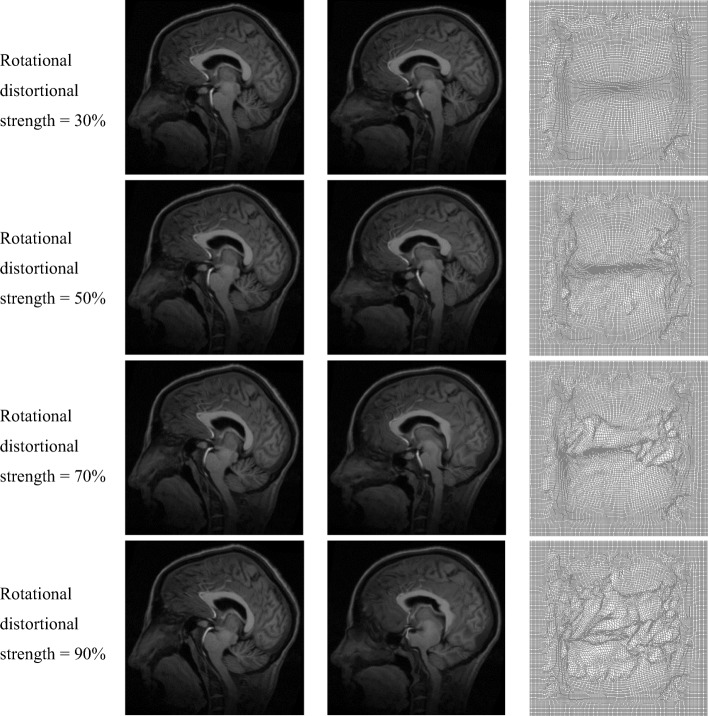
Medical image registration with large deformation produced by rotational distortion. Column 1: moving images produced for rotational distortional strengths of 30–90%, column 2: corresponding registration results, and column 3: corresponding final deformation fields

Medical image registration with large deformation produced by rotational distortion was performed using our proposed method, as shown in Fig. 5. Column 1 show moving images produced with rotational distortional strengths of 30–90%, column 2 shows the corresponding registration result, and column 3 shows the corresponding final deformation field. Table 3 shows the quantitative analysis result; the normalized cross-correlation coefficient and structural similarity decreased and the mean square error and the consumed time gradually increased with an increase in the deformation field strength.

**Table 3:** Evaluation of registration result (rotational distortion deformation)

Rotational Distortional Strength	MSE/(A.U.)	NCC/(A.U.)	Structural Similarity/(A.U.)
30%	531.6939	0.9991	0.9950
50%	1763.9	0.9898	0.9479
70%	2948.6	0.9717	0.9107
90%	5046.0	0.9169	0.8136

**Fig. 6:**
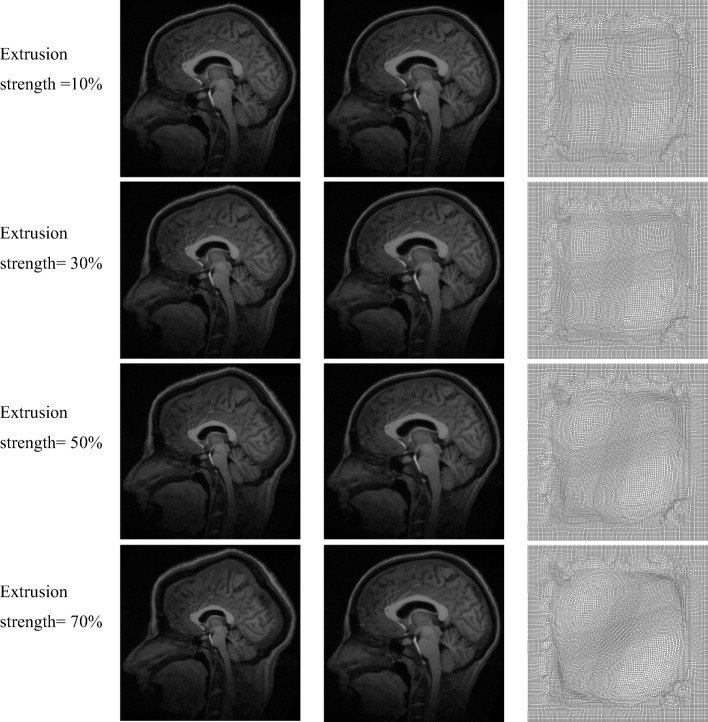
Medical image registration with large deformation produced by extrusion. Column 1: moving images produced for extrusion strengths of 10–70%, column 2: corresponding registration results, and column 3: corresponding final deformation fields

Figure 6 shows the result for large deformation produced by extrusion. Column 1 show moving images produced for extrusion strengths of 10–70%, column 2 shows the corresponding registration result, and column 3 shows the corresponding final deformation field. Table 4 shows the quantitative analysis result; the normalized cross-correlation coefficient, structural similarity, and mean square error basically remained stable and the consumed time gradually increased with an increase in the deformation field strength. Therefore, medical image registration with large deformation produced by both rotational distortion and extrusion can be performed efficiently.

**Table 4:** Evaluation of registration result (extrusion deformation)

Extrusion Strength	MSE/(A.U.)	NCC/(A.U.)	Structural Similarity/(A.U.)
10%	543.2	0.9991	0.9940
30%	545.1	0.9991	0.9944
50%	529.8	0.9991	0.9949
70%	559.4	0.9990	0.9946

### Clinical application

**Fig. 7:**
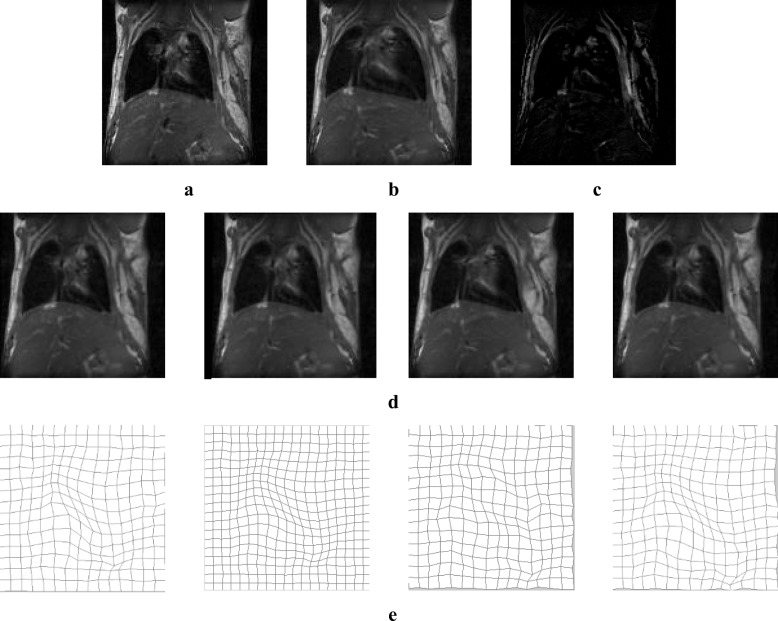
Registration result of pulmonary image: (**a**) reference image; (**b**) moving image; (**c**) initial difference between reference and moving images; (**d**) registration results obtained using our method, diffeomorphic demons, additive demons, and active demons, respectively, from left to right; and (**e**) final deformation field

Pulmonary medical images of the same individual show large deformations under different respiration states, and therefore, non-rigid registration of such images remains challenging. Thoracic images were selected from DIR-Lab (http://www.DIR-lab.com). DIR-Lab provides 12 cases, each of which contains a thoracic image with six phases. Slices with inhale and exhale phases were used as reference and moving images with large deformation. Figure 7 shows the registration result of pulmonary images. The deformation field obtained using our method was smooth and topologically invariant. Table 5 shows the quantitative analysis of the evaluation indexes for thoracic image registration with large deformation; the normalized cross-correlation coefficient and structural similarity are the highest and the mean square error is the lowest; and the evaluation indexes obtained using our method were the closest to the standard value. The registration and statistical results indicate that our method can be clinically applied to the non-rigid registration of pulmonary images with large deformation.

**Table 5:** Evaluation of registration result (lung)

Experiment Method	MSE/(A.U.)	NCC/(A.U.)	Structural Similarity/(A.U.)
our method	1371.9	0.9685	0.7911
diffeomorphic demons	1427.4	0.9678	0.7847
additive demons	1409.3	0.9385	0.7088
active demons	1401.8	0.9377	0.7043
standard result	1002.1	0.9874	0.8210

### Analysis of different driving forces

**Fig. 8:**
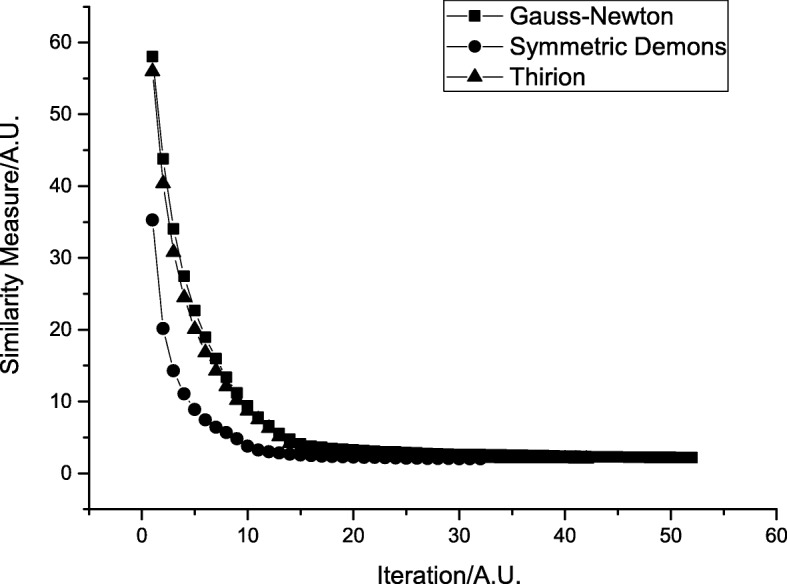
Comparison of three different demons driving forces

Thirion, Gauss-Newton, and symmetric driving forces were used, and the displacement field was defined differently. Figure 8 shows the variation of the energy function *E(t)*; the convergence is the fastest and the similarity measure based on energy function is the lowest with symmetric driving forces. Table 6 shows the quantitative analysis; the symmetric driving force shows the best performance.

**Table 6:** Evaluation of registration result based on different driving forces

Experiment Method	MSE/(A.U.)	NCC/(A.U.)	Structural Similarity/(A.U.)
Thirion	514.79	0.9993	0.9948
Gauss-Newton	476.1	0.9993	0.9948
Symmetric Demons	465.0	0.9994	0.9952

### Influence of parameters on registration result

The Gaussian filter, deformation updating step-length, resolution layer, and convergence condition were used as experimental parameters. The resolution layer can only influence the registration speed, and the convergence condition can influence the registration accuracy. Table 7 shows details about the deformation updating step-length; the registration accuracy remained stable and the consumed time decreased with an increase in the step-length from 0.8 to 2.0.

**Table 7:** Influence of deformation updating step-length *σ*_*x*_ on registration accuracy

Registration Accuracy	*σ*_*x*_				
	2.0	1.5	1.2	1.0	0.8
MSE/(A.U.)	464.81	479.2040	485.8915	508.2908	522.31
NCC/(A.U.)	0.9993	0.9993	0.9992	0.9992	0.9991
Structural Similarity/(A.U.)	0.9953	0.9949	0.9949	0.9943	0.9939
Time Consuming/(s)	136.46	150.37	193.94	222.06	287.1

## Discussion

To verify the superiority of our method, synthetic images and the same modality MRI and CT images were used to perform non-rigid image registration with large deformation. Figures 3(e) and 4(e) show the obtained deformation fields for MRI and CT images. The edge and detail information of final deformation field obtained using our method are more accurate and reasonable than those obtained using active demons and additive demons. The diffeomorphism strategy was used to guarantee smooth and reversible deformation; by contrast, active demons and additive demons produce unreasonable deformation during registration. This conclusion agrees with that reported previously [7, 8]. Tables 1 and 2 show quantitative analysis results for the same modality MRI and CT image registration, respectively; it is seen that the mean square error is the lowest and normalized cross-correlation and structural similarity are the highest when using the proposed method. Therefore, the proposed method is superior to active demons, additive demons, and diffeomorphic demons. Large deformation was simulated by two types of free transform, rotational distortion and extrusion, and the deformation field was determined by the transform strength. The same modality medical image registration with large deformation was performed successfully, as seen from the registration results in Figs. 5 and 6 and the quantitative analysis results in Tables 3 and 4.

The influence of the deformation updating step-length on registration result was analysed. The result shows that non-rigid registration with large deformation can be performed successfully and that the evaluation indexes remain stable when the update is within a reasonable range. The registration speed accelerates with an increase in the step-length update, and the proposed algorithm is robust. The influence of driving forces on the registration result was also analysed; the result shows that the convergence of the proposed algorithm is the fastest and that the evaluation indexes are the most perfect with symmetric driving force. The finding for the driving force is consistent with those reported previously [5, 7]. A similarity energy function based on the grey value was designed, and the termination condition was set according to the variation of the energy function. Furthermore, iterations could be stopped adaptively, and therefore, the registration speed was accelerated. By contrast, when implementing active demons and diffeomorphic demons, the number of iterations needs to be set manually, and this greatly influences the registration result. If the number of iterations is insufficient, the best registration result cannot be obtained. Diffeomorphic demons use the diffeomorphic deformation strategy to guarantee smooth and reversible deformation; however, they are time-consuming. By contrast, additive demons consume less time; however, they cannot guarantee smooth and topologically invariant deformation, and they are prone to producing unreasonable deformations. The proposed algorithm can stop iterations automatically, thus greatly accelerating the registration speed and ensuring a smooth and topologically invariant deformation field.

Pulmonary medical images of the same individual under different respiration states show large deformation, and registration of such images is crucial for planning the treatment of thoracic malignancies and quantitatively analysing pulmonary function. Quantitative analyses showed that pulmonary medical images with large deformation were successfully and efficiently registered using our proposed method, and the deformation was smooth and topologically invariant. Therefore, our proposed method is promising for applications in diagnostic pulmonary imaging and radiation oncology.

## Conclusion

This study proposed an adaptive diffeomorphic multi-resolution demons algorithm and solved the problem of the same modality medical image non-rigid registration with large deformation. Synthetic images and the same modality MRI and CT image registration were tested by this method, and quantitative analyses demonstrated the proposed method’s superiority. Medical images with large deformation were produced by rotational distortion and extrusion transform, registration result and quantitative analyses demonstrated that image registration with large deformation could be performed successfully and efficiently using our method. Quantitative analyses under different driving forces demonstrated that our method based on symmetric driving force is the most efficient. The influence of parameters on registration result was analysed indicating that our method is robust. This method can be also applied to pulmonary medical image registration with large deformation, and have an important clinical application value.

## Abbreviations

CSF: Cerebrospinal fluid
CT: Computed tomography
GM: Grey matter
MRI: Magnetic resonance imaging
MSE: Mean square error
NCC: Normalized cross correlation
WM: White matter
